# Assembly-defective Tembusu virus ectopically expressing capsid protein is an approach for live-attenuated flavivirus vaccine development

**DOI:** 10.1038/s41541-022-00468-y

**Published:** 2022-05-12

**Authors:** Yu He, Jiaqi Guo, Xiaoli Wang, Senzhao Zhang, Li Mao, Tao Hu, Mingshu Wang, Renyong Jia, Dekang Zhu, Mafeng Liu, Xinxin Zhao, Qiao Yang, Ying Wu, Shaqiu Zhang, Juan Huang, Sai Mao, Xumin Ou, Qun Gao, Di Sun, Anchun Cheng, Shun Chen

**Affiliations:** 1 Institute of Preventive Veterinary Medicine, Sichuan Agricultural University, 611130 Chengdu, Sichuan China; 2 Research Center of Avian Disease, College of Veterinary Medicine, Sichuan Agricultural University, 611130 Chengdu, Sichuan China; 3 Key Laboratory of Animal Disease and Human Health of Sichuan Province, Sichuan Agricultural University, 611130 Chengdu, Sichuan China

**Keywords:** Live attenuated vaccines, Virology, Viral host response

## Abstract

Live-attenuated vaccines (LAVs) represent a promising approach for flavivirus vaccine development. In the present study, we demonstrated a method for generating flavivirus LAVs based on breaking spatially and temporally regulated C-prM cleavage to disturb the viral assembly process, using an avian flavivirus (Tembusu virus) as the model. Using reverse genetics technology, we successfully generated two recombinant viruses (CQW1-IRES-mC and CQW1-MINI-mC) with bicistronic genomic RNA in which native capsid genes were deleted and instead expressed in the 3’UTR under the control of an internal ribosome entry site (IRES) or minimum IRES. Both viruses showed a significantly attenuated phenotype in vitro due to impaired viral assembly, and the engineered mutations were genetically stable in vitro within ten passages. Importantly, their virulence was also highly attenuated in ducklings and suckling mice and did not cause any overt clinical symptoms or mortality. In addition, a single dose of immunization with any of these mutant viruses could completely protect ducklings from a lethal challenge, and no viremia was detected after immunization and challenge, even though the viruses induced a relatively moderate immune response in terms of the T-lymphocytes proliferative response and the level of neutralization antibodies compared with that obtained with the wild-type virus. Besides, a recombinant virus ectopically expressing the prM-E protein was also generated in the present study, but this virus was too attenuated with severely decreased proliferation. Our results indicated that the use of a recombinant flavivirus that ectopically expresses structural proteins could be an effective and universal method for flavivirus LAVs development.

## Introduction

Flaviviruses are a group of enveloped, positive-sense RNA viruses, and several flaviviruses transmitted by arthropods (i.e., mosquitos and ticks), including dengue virus (DENV), Zika virus (ZIKV), Wet Nile virus (WNV), yellow fever virus (YFV), and Japanese encephalitis virus (JEV), are important human pathogens and have posed a significant threat to global public health in recent decades. The flavivirus genome is ~11 kb in length and contains a single ORF that encodes a polyprotein with a length of 3400 aa; after processing by the NS2B-3 protease and cell host signalase, the genome generates 3 structural proteins (C, prM, and E) and 7 nonstructural proteins (NS1, NS2A/2B, NS3, NS4A/4B, and NS5). The structural proteins form the virion, and the nonstructural proteins are involved in viral replication and assembly, among other functions. According to a recently proposed model for flavivirus virion assembly, the processing of the flaviviral assembly occurs in a closely coordinated manner. It has been well demonstrated that the sequential processing of the polyprotein C-prM spatially and temporally regulates the incorporation of the nucleocapsid (NC) core into budding virions^1^. The N-terminus of the capsid protein (CP) transmembrane anchor is primarily cleaved by the viral NS2B-3 protease on the cytoplasmic side, and the C-terminus is then processed by host cell signalase in the ER lumen^2–5^. The uncoupling of this coordinated process would impair or even abolish infectious virion production but produce more noninfectious subviral particles (SVPs)^3,4^.

Many medically important flaviviruses have been relatively well studied; however, due to a lack of adequate in vivo models, multiple important aspects of flavivirus infection are poorly understood, or the existing data are contradictory. In this respect, flaviviruses that infect nonhuman hosts represent good research models. Some of these viruses, including avian Tembusu virus (TMUV), are important veterinary pathogens. TMUV is an emerging flavivirus that causes ovaritis and lethal encephalitis in ducks and was responsible for a severe outbreak of duck egg-drop syndrome in 2010, which caused a serious economic loss to the poultry industry in China^6^. TMUV has become one of the most important pathogens of poultry and has a wide range of natural host species, including ducks, geese^7^, chickens^8^, and other birds; mosquitoes are considered transmission vectors for TMUV, and several TMUV strains of mosquito origin have been isolated^9–11^. In addition, artificial infection in the laboratory indicates that TMUV shows high neurovirulence in mice^12,13^. These properties make TMUV an ideal model for flavivirus infection because it is easily accessible for the natural infection of hosts that can then be used as animal models. Importantly, flaviviruses that are native to birds (for example, WNV) can infect humans and cause serious diseases. The TMUV genome and antibodies have also been detected in the human population^14,15^, which makes TMUV another flavivirus with zoonotic potential, even though the virus is highly sensitive to mammalian interferon^12^.

In the present study, we hypothesized that disturbing the C-prM cleavage process would obstruct viral assembly and thus achieve the aim of attenuating the virulence of flaviviruses. Using TMUV as a model, we generated a set of recombinant TMUVs that ectopically expressed mature CP or prM-E and in which the native structural proteins were deleted. These viruses showed attenuated growth kinetics in cell culture due to defects in viral assembly. The viruses were also highly attenuated in suckling mice and ducklings and did not cause any clinical symptoms or mortality. We further evaluated the immunoprotective effectiveness of these viruses in ducks, and both humoral and cellular immune responses were stimulated and protected ducklings from infection. Our results provide the indication that ectopically expressing structural proteins is an effective and universal method for the development of live-attenuated vaccines (LAVs) for flaviviruses.

## Results

### Design and recovery of recombinant viruses expressing CP ectopically

**Fig. 1:**
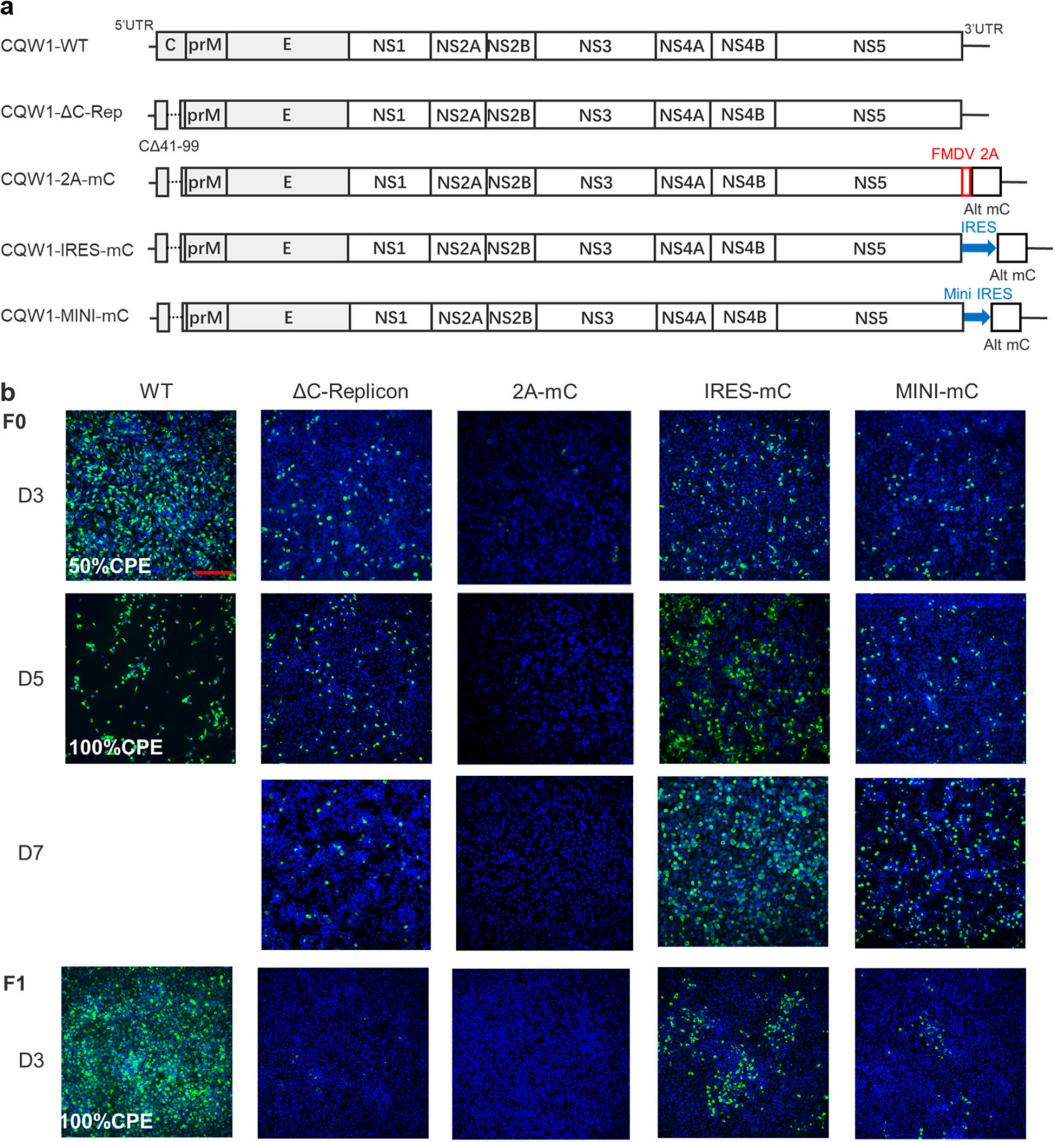
Design and recovery of recombinant TMUV expressing capsid protein ectopically. **a** Schematic diagram for the design of the genomes of CQW1-ΔC-Replicon, CQW1-IRES-mC, CQW1-MINI-mC, and CQW1-2A-mC. **b** Recovery of recombinant virus determined by IFA. BHK-21 cells were transfected with in vitro transcribed RNA (F0). Five days post transfection, the supernatants were harvested for the next round of infection to generate F1 viruses. The cytopathic effect (CPE) is also presented as a percentage if clear CPE is observed. The length of the scale bar (displayed in a red line segment) represents 400 μm.

Previous studies have demonstrated that unlocking the spatially and temporally coordinated cleavage of polyprotein C-prM would impair the viral assembly process and thus lead to premature prM/E-forming SVPs. The present study aimed to assess whether disturbing this process can be an approach for the development of LAVs for flaviviruses. Using avian TMUV as a model, we firstly engineered a mutation into the signalase cleavage site (the last five residues of the CP anchor, PIVAG → PQVQG) to release the limitation of NS2B-3 cleavage upon signalase cleavage. In accordance with previous studies^3,4^, this mutation severely attenuated TMUV proliferation in cell culture by impairing viral assembly and attenuated the virulence of TMUV in duck embryos. However, the viruses with this mutation tended to revert to a highly virulent phenotype after propagation in cell culture. Therefore, we rationally designed a viral genome with ectopically expressed CP in the C-terminus (Fig. 1a). Under these conditions, the release of CP was independent of the processing of the C-prM polyprotein by NS2B-3, but the cleavage of C-prM still occurred normally. According to previous studies on flavivirus reporter viruses, the insertion of markers, particularly large markers, at this position is generally unstable; therefore, we designed three experimental schemes with different sizes of insertions.

After transfection into BHK-21 cells, viral protein expression was assessed by IFA. As shown in Fig. 1b, fluorescent signals were detected in all constructs at 3 days post transfection, but only a very weak signal (less than 1% of positive cells) was observed with CQW1-2A-mC. No obvious signal was observed with CQW1-2A-mC at 5 and 7 days. To confirm the infectivity of these recombinant viruses, the supernatants were harvested at 5 days post transfection and used to infect fresh BHK-21 cells. At 3 days post-infection (dpi), strong fluorescence (100% of positive cells) was detected in the wild-type (WT)-infected cells, and a moderate signal was also detected in the CQW1-IRES-mC- and CQW1-MINI-mC-infected cells, but no signal was observed in the CQW1-ΔC-Rep- and CQW1-2A-mC-infected cells. This result indicated that infectious particles were produced by CQW1-IRES-mC and CQW1-MINI-mC; in addition, although the transfection of CQW1-ΔC-Rep RNA generated visible fluorescence, it did not produce infectious particles, which indicated that the mutation of ΔC41-99 resulted in this mutant becoming a noninfectious replicon. In addition, CQW1-2A-mC is unviable, and the underlying reason will be analyzed in subsequent sections.

### In vitro properties of recombinant viruses expressing CP ectopically

**Fig. 2:**
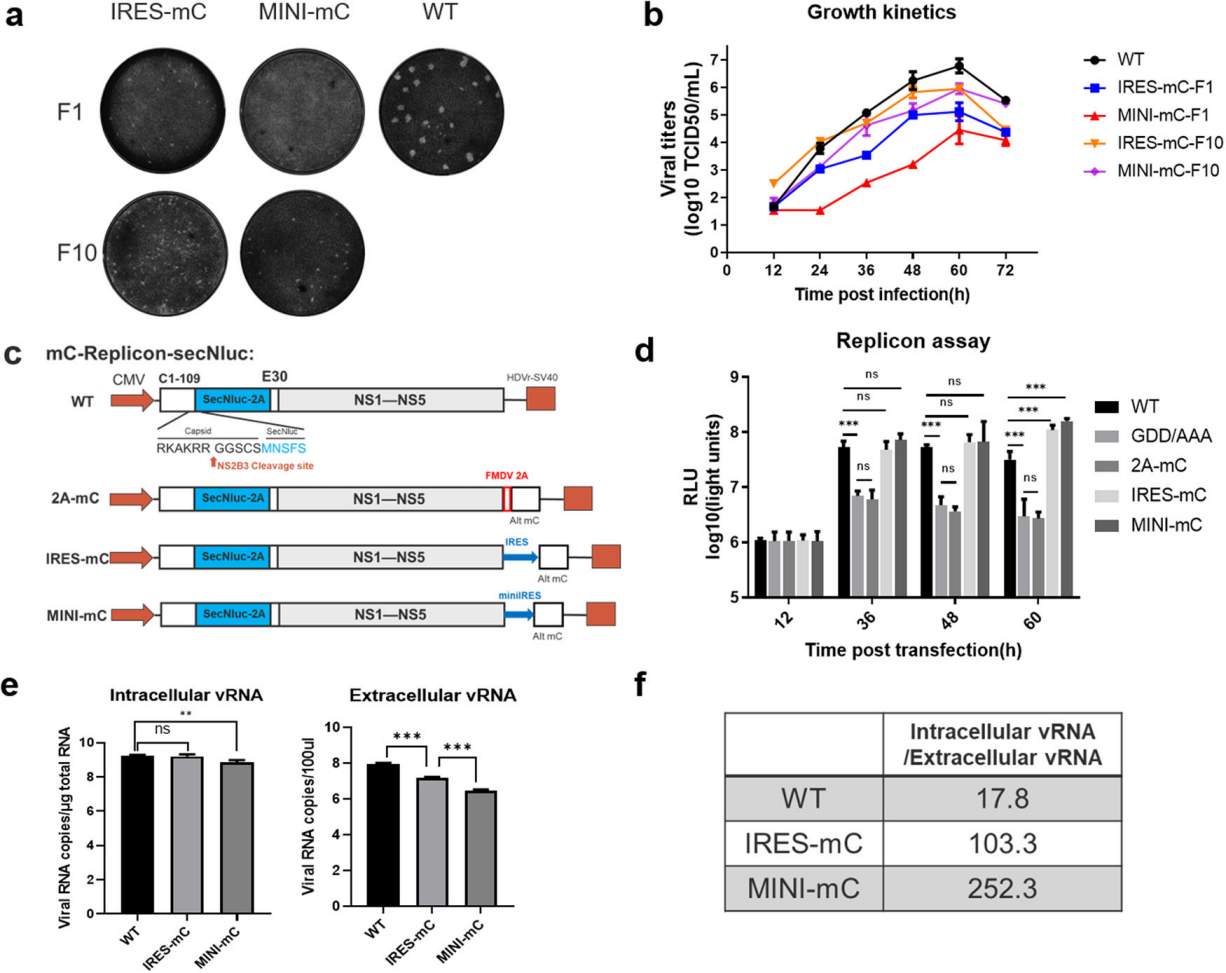
In vitro properties of recombinant viruses ectopically expressing CP. **a** Plaque morphology and **b** growth kinetics of the CQW1-IRES-mC and CQW1-MINI-mC viruses on BHK-21 cells. **c** This panel depicts the DNA-based replicons expressing secretory NLuc (mC-Replicon-SecNLuc) used in panel **d**. Only 1–109 aa of CP and the last 30 residues of the E protein were retained for replicons. A replication-defective replicon (GDD/AAA) with inactivated NS5-GDD motifs was used as a negative control. The effect of these insertions in the 3’UTR on viral RNA replication was verified via a replicon assay. **e** RT–qPCR analysis of intracellular/extracellular vRNA. An equal dose (3000 TCID_50_) of each virus was used to infect BHK-21 cells for 18 h to avoid multiple rounds of infection, and the viral copies in the intracellular or extracellular space were then measured. **f** Intracellular/extracellular vRNA ratios of WT, IRES-mC, and MINI-mC viruses calculated from **e**. Three independent experiments are presented as the means and SEMs; significance was defined by a *p*-value < 0.05 (*p < 0.05; **p < 0.01; ***p < 0.001), and ns indicates no significance.

In the above section, although CQW1-IRES-mC or CQW1-MINI-mC were viable in BHK-21 cells, the viral spread of CQW1-IRES-mC and CQW1-MINI-mC appeared slower than that of the WT virus. Therefore, we evaluated the plaque morphology of CQW1-IRES-mC and CQW1-MINI-mC on BHK-21 cells (Fig. 2a). CQW1-IRES-mC generated significantly smaller plaques than the WT virus, whereas CQW1-MINI-mC did not form visible plaques under these conditions. We then further evaluated the growth kinetics of CQW1-IRES-mC and CQW1-MINI-mC on BHK-21 cells. As shown in Fig. 2b, the proliferation of both CQW1-IRES-mC (with a peak titer of 10^5.12^ TCID_50_/mL) and CQW1-MINI-mC (with a peak titer of 10^4.46^ TCID_50_/mL) was highly attenuated. Both viruses were found to release significantly fewer infectious particles than the WT virus, and the peak titers were more than 46-fold lower than those found for the WT virus (with a peak titer of 10^6.79^ TCID_50_/mL). CQW1-MINI-mC showed particularly attenuated proliferation.

To determine the molecular basis for the attenuation of CQW1-IRES-mC and CQW1-MINI-mC on BHK-21 cells, we first performed replicon assays to verify the effect of the gene cassettes on viral RNA replication (by measuring NanoLuc luciferase to determine viral subgenomic replicon RNA replication) based on the consideration that these insertions are located in the 3’UTR (Fig. 2c). As shown in Fig. 2d, no significant differences were found between the WT replicon and the IRES-mC or MINI-mC-replicon (except for the timepoint of 72 h post transfection), which indicated that the insertion of IRES-mC and MINI-mC had little effect on viral replication. However, the 2A-mC-replicon showed a kinetics curve similar to that of the replication-deficient replicon with the NS5-GDD/AAA mutation, which indicated that 2A-mC, which is fused in frame downstream of the NS5 gene, directly abolished viral replication. This outcome could be due to the extended C-terminus of NS5 interrupting its functions, which makes CQW1-2A-mC RNA unable to produce virions.

It has been well documented that flaviviral assembly is a highly coordinated process, and dysfunction of CP results in increased production of subviral particles (SVPs), which do not contain the NC core, at the expense of infectious particles. Therefore, we examined the assembly efficiency of these two viruses on BHK-21 cells. The total infection time was restricted to 18 h to avoid multiple rounds of infection, and the cells were infected with equal amounts of virus (3000 TCID_50_, MOI = 0.06). After 1 h of incubation, unattached viruses were removed from the cells through washing with PBS. At 18 h post-infection, intracellular and extracellular vRNAs were quantified by quantitative reverse transcription PCR (RT–qPCR). As shown in Fig. 2e, only CQW1-MINI-mC produced slightly (2.3-fold) less intracellular vRNA than the WT virus, and the viral copies of CQW1-IRES-mC were comparable to those of the WT virus; however, the extracellular vRNA of CQW1-IRES-mC and CQW1-MINI-mC were 6.1-fold and 33-fold lower than WT virus, respectively. We then calculated the ratio of intracellular vRNA to extracellular vRNA to measure viral assembly. The results showed that the intracellular RNA/extracellular vRNA ratios derived from the CQW1-IRES-mC and CQW1-MINI-mC viruses were 5.8- and 14.2-fold higher than those derived from the WT virus, respectively (Fig. 2f), which suggested that the mutant virus exhibited more inefficient virion assembly/release. We reasoned that the relative inefficiency of CQW1-MINI-mC could be due to the lower efficiency of the minimum IRES element for translation and ectopic expression of mC compared with the results observed with CQW1-IRES-mC.

Altogether, these data indicated that CQW1-IRES-mC and CQW1-MINI-mC showed attenuated growth phenotypes in vitro and that their viral assembly/release process but not viral RNA replication was impaired.

### Stability of recombinant viruses expressing CP ectopically

We subsequently determined the stability of CQW1-IRES-mC and CQW1-MINI-mC through continuous passage in BHK-21 cells. A more rapid cytopathic effect was observed with these two viruses during continuous passage. After ten passages, the viruses were subjected to plaque assay to determine whether better growth phenotypes were obtained. As shown in Fig. 2a, CQW1-IRES-mC always formed plaques of similar size in the F1 and F10 generations; no clear plaque was observed with CQW1-MINI-mC-F1, but CQW1-MINI-mC-F10 formed distinct plaques that were as large as those formed by CQW1-IRES-mC. In contrast, the WT virus formed significantly larger plaques. We further quantitatively analyzed the infectious virus particle production levels of WT virus and the F10 generation mutant viruses by comparing their growth curves on BHK-21 cells (Fig. 2b). Compared with the F1 generation, CQW1-MINI-mC-F10 produced significantly more infectious particles, and reached a peak titer of 10^6^ TCID_50_/mL, which was 31.6-fold higher than its F1 virus; CQW1-IRES-mC-F10 showed a moderate increase in the number of infectious particles compared with the F1 generation, reached a 6.9-fold higher peak titer (10^6^ TCID_50_/mL) in comparison to its F1 generation. The F10 generation of both mutant viruses was then subjected to whole genome sequencing to identify the adaptive mutations that emerged during continuous passage (Table 1). Five mutations and three mutations were observed in CQW1-IRES-mC-F10 and CQW1-MINI-mC-F10, respectively, but the engineered mutations stably existed.

**Table 1:** Continuous passages of CQW1-IRES-mC and CQW1-MINI-mC on BHK-21 cells.

CQW1-IRES-mC-F10			CQW1-MINI-mC-F10		
Location	Nucleotide position	Amino acid change	Location	Nucleotide position	Amino acid change
E	A2131G	Q392R	NS3	A6303G	T572A
NS3	T4906C	V106A	NS5	A7708G	Q18R
NS5	A7708G	Q18R	3’UTR	C10588T	—
	G9582A	G643R			
	C9707T	H684^a^			

^a^Indicates silent mutation.

### In vivo virulence of CQW1-IRES-mC and CQW1-MINI-mC

**Fig. 3:**
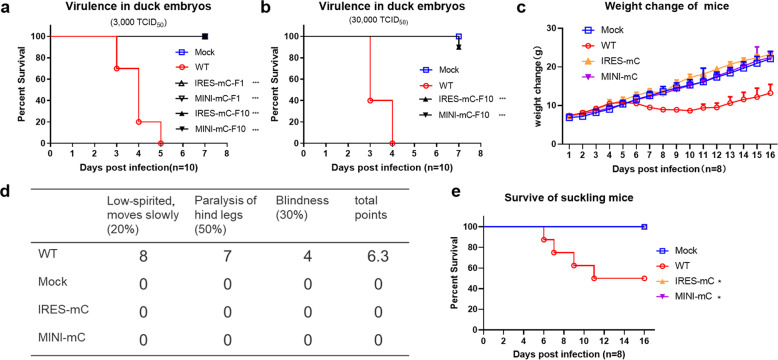
In vivo virulence of CQW1-IRES-mC and CQW1-MINI-mC. Virulence of the CQW1-IRES-mC and CQW1-MINI-mC viruses in 9-day-old duck embryos (*n* = 10) via allantoic cavity infection at a dose of **a** 3000 TCID_50_ or **b** 30,000 TCID_50_. Neurovirulence was determined in 14-day suckling Kunming mice (*n* = 8) by the intracerebral inoculation of CQW1-IRES-mC or CQW1-MINI-mC viruses at a dose of 30 µL containing 10^4.23^ TCID_50_. The weight changes (**c**), clinical symptoms (**d**), and mortality (**e**) of the infected mice were then recorded. The statistical significance of the survival of the mice was analyzed using a survival curve and the log-rank (Mantel–Cox) test; significance was defined by a *p*-value < 0.05 (*p < 0.05; **p < 0.01; ***p < 0.001), ns indicates no significance.

To assess the ectopic expression of CP on viral virulence, we first evaluated the virulence of CQW1-IRES-mC and CQW1-MINI-mC on duck embryos, a model somewhere between in vitro and in vivo. When infected at a dose of 3000 TCID_50,_ 10 embryos infected with the WT virus succumbed within 5 days, which indicated that the WT virus was highly virulent to duck embryos, but no embryos in the CQW1-IRES-mC-F1- and CQW1-MINI-mC-F1-infected groups succumbed during the observation period (Fig. 3a), which indicated that the mutant viruses ectopically expressing CP were highly attenuated in duck embryos. To determine whether the virulence of CQW1-IRES-mC-F10 and CQW1-MINI-mC-F10, which exhibited better growth phenotypes in vitro, reverted to the WT level, duck embryos were infected with these viruses at an equal dose, but no embryos succumbed during the experiment. Even though a 10-fold dose of F10 virus was used for infection, 90% of duck embryos infected with the CQW1-IRES-mC of CQW1-MINI-mC virus survived; in contrast, all duck embryos succumbed within 4 days after WT virus infection (Fig. 3b). Overall, these data indicated that the CQW1-IRES-mC and CQW1-MINI-mC viruses were highly attenuated in duck embryos; even after 10 continuous passages on cells, their virulence did not revert to the WT levels.

We then further determined the attenuation of the neurovirulence of the CQW1-IRES-mC and CQW1-MINI-mC viruses in a suckling mouse model. As expected, the mice belonging to the mock-infected group did not show any clinical symptoms. All suckling mice in the WT virus-infected group started to exhibit various degrees of clinical signs at 5 dpi, including depression, blindness, and paralysis of the hind leg (Fig. 3d). Four out of 8 mice in the WT virus-infected group succumbed to encephalitis during the trial (Fig. 3e); in accordance to this outcome, the body weight of the mice belonging to the WT-infected group declined starting at Day 6 and then recovered their weight on Day 11 to a weight that was significantly lower than that of the mice belonging to the mock group on Day 7 and afterward (Fig. 3c). In contrast, neither the CQW1-IRES-mC- nor the CQW1-MINI-mC-infected groups showed any symptoms, the curves of their body weight gain were undistinguishable from those of the mock group, and no mice succumbed (Fig. 3c–e). Altogether, these data indicated that the neurovirulence of both the CQW1-IRES-mC and CQW1-MINI-mC viruses was significantly attenuated in suckling mice.

### Immune responses stimulated by CQW1-IRES-mC and CQW1-MINI-mC

**Fig. 4:**
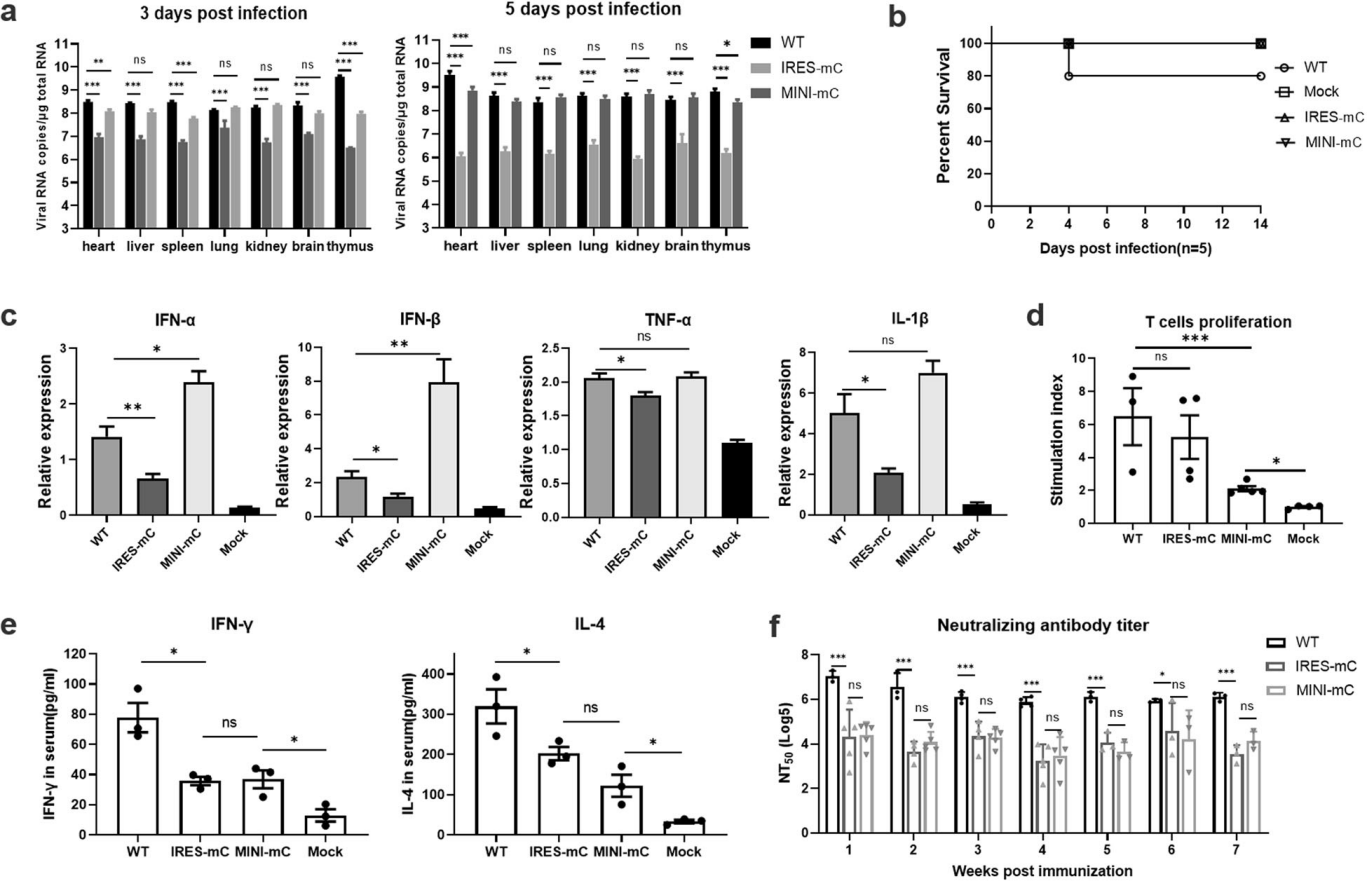
Immune responses stimulated by the CQW1-IRES-mC and CQW1-MINI-mC viruses. Twenty-five-day-old ducks were infected with the WT virus, CQW1-IRES-mC, or CQW1-MINI-mC virus at a dose of 10^5^ TCID_50_. The mock group was treated with DMEM. **a** The vRNA loads in the heart, liver, spleen, lung, kidney, brain and thymus of the ducks at 3 and 5 dpi were detected by RT–qPCR (*n* = 3). **b** Survival rate of 25-day-old ducks after infection (*n* = 5). **c** The relative mRNA expression of IFN-α, IFN-β, TNF-α, and IL-1β in the spleen was detected at 5 dpi (*n* = 3). **d** Duck peripheral T-lymphocyte proliferative response to TMUV infection. At 14 dpi, blood T lymphocytes were isolated and cultured in vitro and then specifically stimulated to proliferate by TMUV or purified recombinant truncated E protein. After 36 h of cell culture at 37 °C, cell proliferation was detected using a CCK-8 kit. **e** At 14 dpi, the serum levels of IFN-γ and IL-4 in the ducks were determined by ELISA. **f** The neutralization antibody levels in the serum were determined by PRNT. The data are presented as the means and SEMs; significance was defined by *p < 0.05; **p < 0.01; ***p < 0.001, and ns indicates no significance.

To better understand the characteristics of CQW1-IRES-mC and CQW1-MINI-mC in vivo, we compared the levels of immunogenicity of CQW1-IRES-mC and CQW1-MINI-mC in ducklings. Twenty-five-day-old ducklings were infected with WT virus or mutant viruses at a dose of 10^5^ TCID_50_. The vRNA load in various organs was measured by RT–qPCR (Fig. 4a). Unexpectedly, at 3 dpi, the vRNA load of CQW1-MINI-mC showed a high replication level (reached 10^8^ copies/μg total RNA), similar to the results obtained with the WT virus, in most organs with the exception of the heart, thymus and spleen, and the vRNA load of both the WT virus and the CQW1-MINI-mC virus was further increased by ~10-fold at 5 dpi. However, the viral loads of CQW1-IRES-mC in almost all tested organs were lower than that of WT virus by approximately an order of magnitude, at 3 dpi; and decreased to an extremely low level at 5 dpi. Only 1 duckling infected with the WT virus exhibited neurological signs and succumbed at 4 dpi; no duckling succumbed after infection with the CQW1-IRES-mC or CQW1-MINI-mC virus or showed neurological signs over the duration of the experiment (Fig. 4b). Furthermore, viral RNA isolated from tissues of CQW1-IRES-mC- and CQW1-MINI-mC-infected ducks was further analyzed by RT–PCR followed by sequencing, with excluded the possibility of WT virus contamination.

To compare the abilities of WT, CQW1-IRES-mC and CQW1-MINI-mC viruses to induce innate cytokine responses, we measured the relative mRNA levels of IFN-α, IFN-β, TNF-α, and IL-1β in spleen samples. Both the CQW1-IRES-mC and CQW1-MINI-mC virus infections induced a similar innate immune response pattern to that obtained with the WT virus. All of the cytokines were upregulated in all virus-infected groups, particularly the group infected with the CQW1-MINI-mC virus, which induced higher expression of cytokines, including IFN-α, IFN-β, and IL-1β, than the WT virus (Fig. 4c). In contrast, CQW1-IRES-mC infection stimulated relatively moderate upregulation of these cytokines compared with that observed with the WT virus.

We further tested whether cellular immunity can be activated by CQW1-IRES-mC and CQW1-MINI-mC virus infection. At 14 dpi, ducks were bled to determine the peripheral T-lymphocyte proliferative response to TMUV infection (Fig. 4d), and the levels of IFN-γ (Th1-type cytokine) and IL-4 (Th2-type cytokine) in serum (Fig. 4e) were also determined. After stimulation, the T lymphocytes from all virus-infected ducks produced significantly higher levels of proliferation than those from the mock group (Dulbecco’s modified Eagle medium (DMEM)-treated). The CQW1-IRES-mC group showed a high level of proliferation comparable to that found with the WT group, and the CQW1-MINI-mC group exhibited a moderate level of proliferation, only one third level of WT group. Consistently, the CQW1-IRES-mC- and CQW1-MINI-mC-infected ducks produced significantly higher levels of IFN-γ and IL-4 than those belonging to the mock group. These data indicated that infection with the CQW1-IRES-mC or CQW1-MINI-mC virus can activate cellular immunity in ducks. As neutralizing antibodies are essential for protecting the host from flavivirus infection, we then determined the presence of neutralizing antibodies in the serum of ducks infected with the CQW1-IRES-mC or CQW1-MINI-mC virus. As shown in Fig. 4f, similar to cellular immunity, both CQW1-IRES-mC and CQW1-MINI-mC induced detectable levels of neutralizing antibodies as early as 1 week post-infection, and the neutralizing antibodies lasted at least 7 weeks, but the levels of antibodies were ~20–100-fold lower than those obtained with the WT virus.

Altogether, these data indicated that both CQW1-IRES-mC and CQW1-MINI-mC were highly attenuated in vivo but elicited both innate immune and adaptive immune responses, although the levels of immune responses induced by CQW1-IRES-mC and CQW1-MINI-mC virus infection were less efficient than those induced by WT virus infection.

### Single-dose immunization with recombinant viruses expressing CP protected ducklings from lethal challenge with an epidemic TMUV

**Fig. 5:**
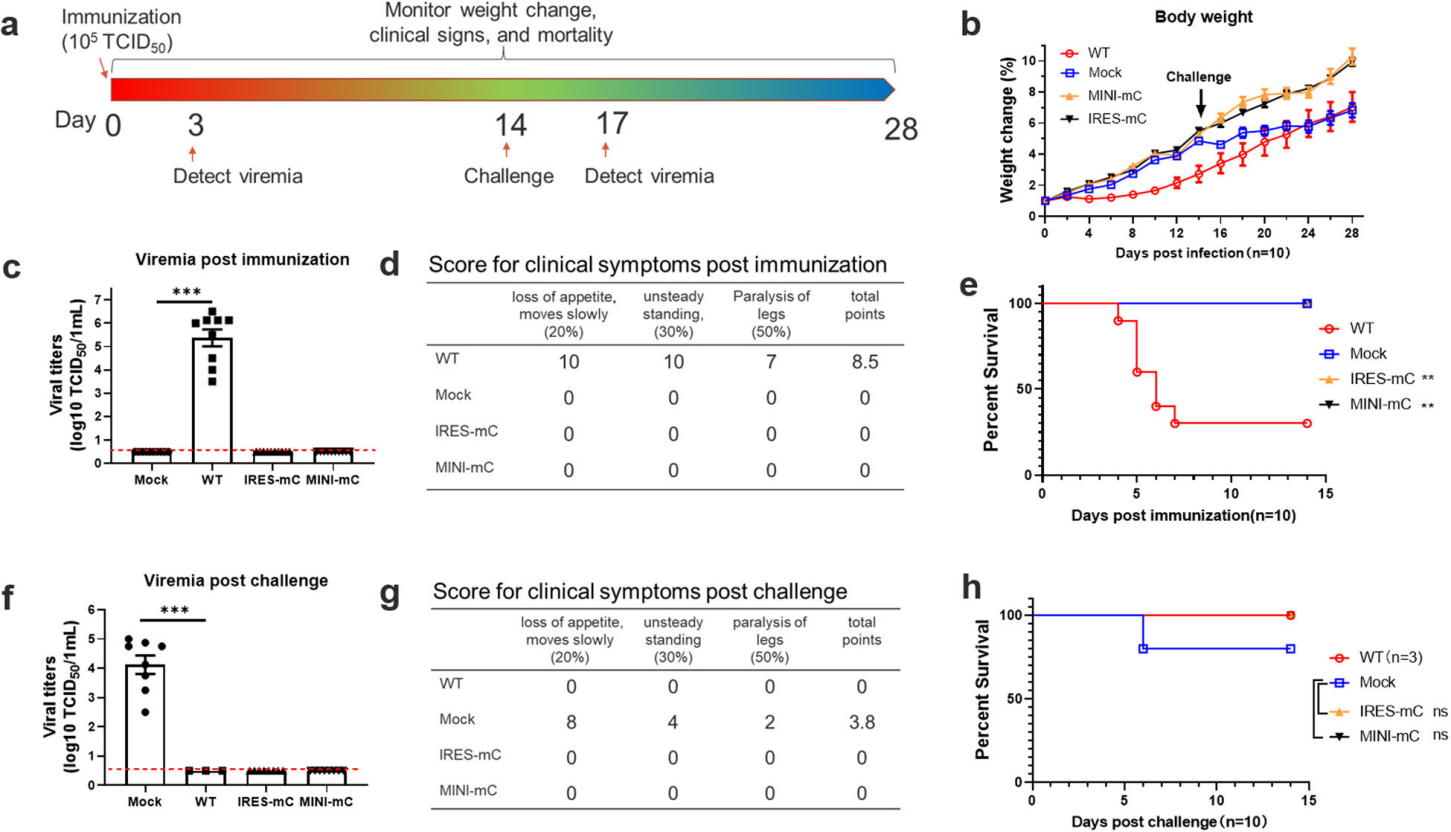
Single-dose immunization with the CQW1-IRES-mC and CQW1-MINI-mC viruses protected ducklings from lethal challenge by an epidemic TMUV. **a** Experimental design of the animal experiment. Five-day-old ducklings (*n* = 10) were intramuscularly injected with the WT virus, CQW1-IRES-mC mutant virus or CQW1-MINI-mC mutant virus at a dose of 10^5^ TCID_50_. The mock group was treated with DMEM. At 14 days post-immunization, the surviving ducklings were challenged with virulent TMUV. **b** Weight changes of the ducks after immunization (days 1–14) and challenge (days 15–28). **c** Viremia after immunization. **d** Clinical symptoms after immunization. For each item, a score of 1 corresponded to 1 duck exhibiting the corresponding symptom. The total point was calculated as 20% * Item 1 + 30% * Item 2 + 50% * Item 3. **e** Percentage of survival after immunization. **f** Viremia after challenge. **g** Clinical symptoms after challenge. **h** Percentage of surviving ducklings after challenge. The data are presented as the means and SEMs; significance was defined by *p < 0.05; **p < 0.01; ***p < 0.001, and ns indicates no significance.

We further evaluated the virulence and immunogenicity of CQW1-IRES-mC and CQW1-MINI-mC in ducklings. Fig. 5a depicts the experimental design. As older ducklings can be more resistant to avian TMUV infection, 5-day-old ducklings, a more sensitive animal model, were used to assess the virulence of the CQW1-IRES-mC and CQW1-MINI-mC viruses in ducklings. Ducklings were first immunized with the WT virus, CQW1-IRES-mC virus or CQW1-MINI-mC virus at an equal dose of 10^5^ TCID_50_. The weight change, clinical symptoms, and mortality were monitored for 14 days. Compared with the mock (DMEM)-infected group, no difference in weight change was observed in the CQW1-IRES-mC virus- and CQW1-MINI-mC virus-infected groups, whereas the WT virus-infected ducklings exhibited significantly reduced body weight gain (Fig. 5b). At 3 dpi, the WT virus-infected ducklings produced robust viremia with a titer of 10^5.375^ TCID_50_/mL, but no detectable viremia was detected in the CQW1-IRES-mC virus- and CQW1-MINI-mC virus-infected ducklings (Fig. 5c). In addition, the WT virus-infected ducklings developed a series of clinical symptoms, including loss of appetite, depression, unsteady standing and even paralysis of the legs (Fig. 5d), and 70% of ducklings (n = 7/10) succumbed to WT virus infection (Fig. 5e). In contrast, neither morbidity nor mortality was observed among the CQW1-IRES-mC virus- or CQW1-MINI-mC virus-infected ducklings. Collectively, the results demonstrated that the CQW1-IRES-mC and CQW1-MINI-mC viruses are highly attenuated in their natural hosts (ducks).

We then verified whether the ducklings immunized with CQW1-IRES-mC or CQW1-MINI-mC virus can be protected from a lethal challenge of TMUV. At 14 days post-immunization, ducklings were challenged with the epidemic avian TMUV strain CHN-YC at a dose of 2 × 10^6^ TCID_50_ in 200 μL (Fig. 5a). After the challenge, the ducklings were monitored as described above. At 3 days post-challenge, the mock-immunized ducklings developed obvious viremia (10^4.125^ TCID50/mL), but the CQW1-IRES-mC virus- or CQW1-MINI-mC virus-immunized animals did not exhibit any detectable viremia (Fig. 5f). Moreover, after challenge, the mock-immunized animals showed a significant slow weight gain starting from Day 2 (Fig. 5b) and exhibited clinical symptoms, including loss of appetite, depression and unsteady standing (Fig. 5g), and 20% of ducklings even developed paralysis of the legs and mortality (Fig. 5h). In comparison, the ducklings belonging to the WT virus-, CQW1-IRES-mC virus- and CQW1-MINI-mC virus-immunized groups did not exhibit any obvious symptoms or mortality. Therefore, these data indicated that a single-dose immunization of CQW1-IRES-mC or CQW1-MINI-mC virus could protect ducklings from lethal challenge with a virulent TMUV.

### Recombinant virus ectopically expressing prM-E proteins

**Fig. 6:**
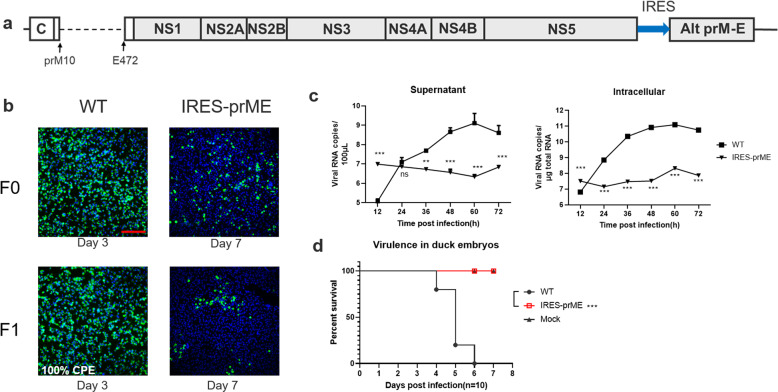
Recombinant TMUV ectopically expressing prM-E protein. **a** Schematic diagram of the construction of CQW1-IRES-prME ectopically expressing prM-E protein. **b** Recovery of CQW1-IRES-prME confirmed by IFA. BHK-21 cells were transfected with pACNR-FL-CQW1-IRES-prME plasmids (F0), and 7 dpi, the supernatants were harvested for the next generation to verify viral infectivity. The length of the scale bar (displayed in a red line segment) represents 400 μm. **c** Growth kinetics of CQW1-IRES-prME measured by RT–qPCR. **d** Virulence of the CQW1-IRES-prME viruses in 9-day-old duck embryos. The data are presented as the means and SEMs; significance was defined by *p < 0.05; **p < 0.01; ***p < 0.001, and ns indicates no significance.

Based on the above-described data, we wondered whether other viral proteins can be supplied in trans and ectopically expressed to support viral replication. It has been well demonstrated that flavivirus prM-E protein could be trans-complemented, and our previous study also demonstrated that trans-complemented prM-E proteins could package replicon RNA to form single-round infectious particles^16^. Therefore, we tested whether the ectopic expression of prM-E protein could generate infectious viruses. Fig. 6a depicts the design of the recombinant virus genome, in which the prM-E gene was deleted in situ and instead expressed in the 3’UTR under the control of an IRES element. Seven days post transfection, the supernatant (F0 virus) from the transfected cells was harvested for the next generation (F1), and a weak positive signal of E protein was detected by IFA in both the F0 and F1 generations of CQW1-IRES-prME (Fig. 6b), which indicated that the recombinant viruses were successfully rescued. However, it appears that this mutant virus was severely attenuated in cell culture, and clear CPE did not occur even at more than 12 days post transfection with the F0 virus and 9 days with the F1 virus. Therefore, we determined the growth kinetics of the F1 virus of CQW1-IRES-prME on BHK-21 cells by RT–qPCR. As shown in Fig. 6c, the viral RNA replication of CQW1-IRES-prME was significantly impaired compared with that of the WT virus. We further assessed its virulence in its natural host (duck embryos) at a dose of 1000 TCID_50_, and no duck embryos succumbed due to CQW1-IRES-prME virus infection (Fig. 6d). In addition, CQW1-IRES-prME was quite stable and did not revert to the WT genotype after 5 passages in BHK-21 cells. However, this virus only kept producing a very low level of viral titer, even at the F5 and later generations. Hence, considering its overattenuated phenotype in cell cultures, we did not further assess the properties of this virus in vivo.

## Discussion

Flaviviruses pose a significant threat to global public health, and a recent example is the outbreak of ZIKV throughout the Americas^17^. Currently, there is no specific medical treatment against diseases caused by flaviviruses; hence, safe and efficacious vaccines are of great importance for preventing viral infection and spread. LAVs represent a feasible and attractive approach for combating flavivirus infection, and flaviviral LAVs are characterized by their high efficiency in seroconversion and the long-term protection they provide to animals. However, the classical methods to generate LAVs are time-consuming and laborious; the need for continuous passaging to accumulate various mutations makes the use of this technique impractical for responding to public health emergencies. Thus, using reverse genetics technology to design the viral genome with the aim of obtaining a desired phenotype within a short time is one of the ideal methods. Although live chimera virus is an alternative method (e.g., by replacing the YF17D prM-E gene with other flavivirus prM-E genes), the virus still sporadically fails to attenuate^18^. In the present study, we used zoonotic-potential TMUV as a research model that is convenient for evaluating its in vivo aspects in its natural host (duck). We demonstrated that ectopically expressing the structural proteins of flaviviruses is an effective method for attenuating viral virulence in vivo and has the potential to generate LAVs within a few days. Our results showed that CQW1-IRES-mC/CQW1-MINI-mC was highly attenuated in 5-day-old ducklings and did not produce detectable viremia at 3 dpi or any symptoms. A single immunization with CQW1-IRES-mC or CQW1-MINI-mC was sufficient to prevent ducklings from a lethal challenge, and no infectious challenge virus was detected in serum, indicating a highly efficient protective immunity was induced. These results showed that this method represents a potential approach for the development of LAVs for flaviviruses.

**Fig. 7:**
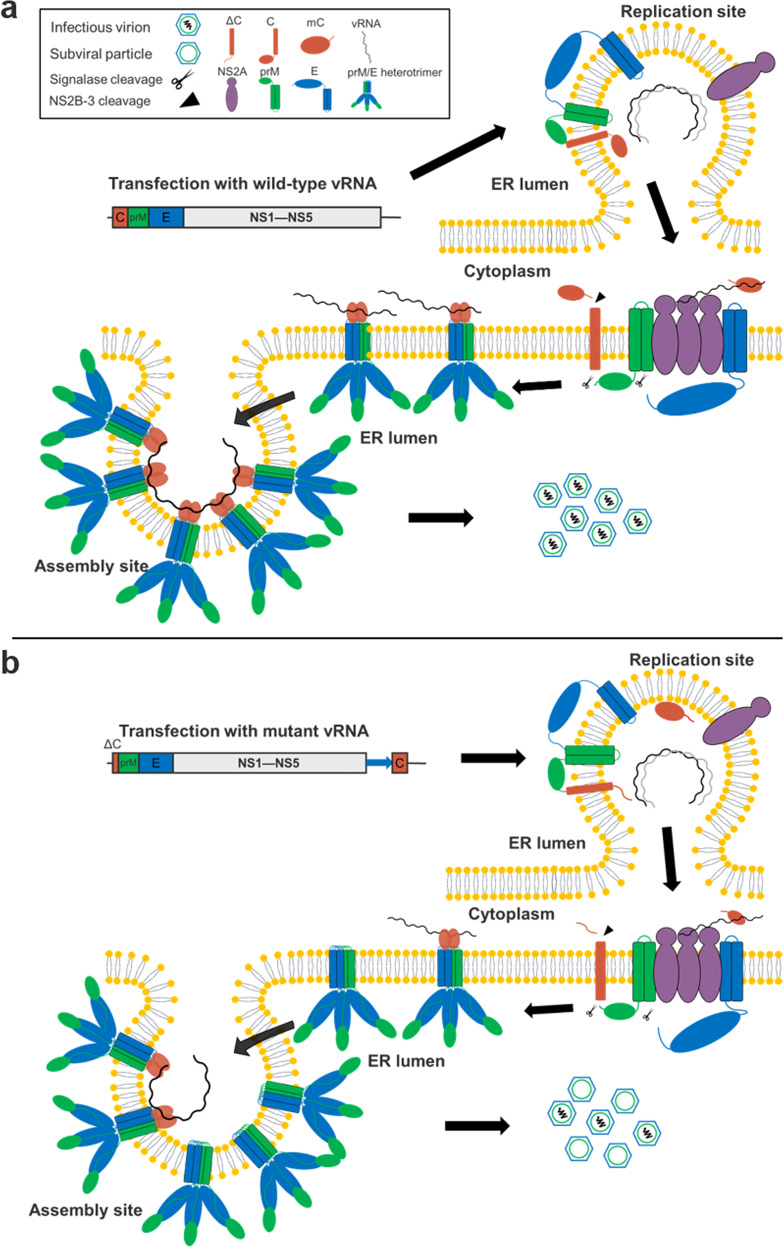
Model of mutant virus ectopically expressing mature capsid protein. **a** Continuous cleavage of flavivirus C-prM polyprotein based on a recently proposed model for the flavivirus assembly process^1^. After translation, the C-prM-E polyprotein spans the ER membrane via each transmembrane anchor sequence. C-prM-E and vRNA are recruited to the assembly site by NS2A, and C-prM then undergoes continuous cleavage by NS2B-3 and signalase to prevent the secretion of subviral particles containing only prM/E. **b** Recombinant viruses ectopically expressing mature capsid protein. The functional capsid protein is not transported to the assembly site when prM-E is recruited by NS2A, which results in the nucleocapsid not being efficiently incorporated into budding virions and secreting more SVPs.

Although the flaviviral assembly step is one of the least understood processes in the viral lifecycle, important advances have been made toward our understanding of this process in recent years (for review, see^1^). It has been well demonstrated that flavivirus virion assembly is a tightly regulated process; a classic example is the coordinated two-step proteolytic process at the C-prM junction^3,19^. According to the recently proposed model for viral assembly^1,20^, C-prM polyprotein cleavage is spatially and temporally regulated. At the late stage of the flavivirus infection cycle, the NS2A protein recruits the C-prM-E polyprotein and NS2B-3 protease to the assembly site. The C-prM polyprotein is then subjected to sequential cleavage by the NS2B-3 protease and signalase, and signalase cleavage remains inefficient until NS2B-3 cleavage occurs to prevent premature prM/E from forming SVPs^1^. Subsequently, dimerized CP associates with vRNA to form NC, and NC is ultimately incorporated into budding virions^1,20^. In the present study, in these bicistronic RNA genomes, the translation of ectopically expressed mC was independent of native polyprotein translation, which resulted in the release of mC ahead of time, and functional mC could not be efficiently recruited to the assembly site along with the prM-E protein to form NCs, which broke the spatially and temporally regulated viral assembly process, even though two-step cleavage of the C-anchor still occurred (Fig. 7). However, less efficient recruitment of mC to the assembly site still enabled sufficient replication of the bicistronic viruses in vitro, yielded sufficient virus titers for vaccine production and in vivo induced protective immunity in vaccinated animals but did not cause any disease. For the mutant virus expressing prM-E ectopically, the independent translation of prM-E would directly result in premature viral assembly and budding without delay in an NC-independent manner to form SVPs secreted out to cells, which could be responsible for the overly attenuated phenotype of CQW1-IRES-prME. Thus, defective assembly is the molecular determinant for the attenuated phenotypes of the mutants generated in the present study (Fig. 7), and our results experimentally prove that the integrality of the C-prM polyprotein for cleavage is important for correct viral assembly. Theoretically, all of the viral proteins that could be supplemented in trans could support virion production by ectopic expression. In addition to structural proteins, flavivirus NS1 is also an alternative target^21^. As the time order of the posttranslational process of flavivirus polyproteins is not well understood, using this approach help us understand the effect of the posttranslational process of NS1 on viral lifecycles.

Based on the theoretical basis described above, this approach has two benefits. (1) Universality among flaviviruses: because all flaviviruses have a similar genomic structure and assembly mechanism, the ectopic expression of structural proteins could be used for other flaviviruses, not only TMUV. (2) Authentic structural proteins: This approach does not involve mutations in structural proteins and maintains the primary structure of viral proteins, and thus, this approach could be beneficial for inducing specific immune responses. In addition, the molecular determinant of their attenuated phenotypes has been resolved, and rapid preparation using reverse genetics tools also has advantages. The only concern for this method is unforeseeable RNA recombination events, which could contribute to the generation of a WT genome. However, in the present study, we re-edited the codons of ectopically expressed genes, and CQW1-IRES-mC/CQW1-MINI-mC exhibited satisfactory stability in vitro. In addition, a previous study also proposed that intergenomic recombination most likely does not represent a major risk for flavivirus LAVs^22^. In addition, this method could also be combined with other mutations (e.g., substitution of E-T367K or E-M304R for TMUV^23,24^) in viral proteins to further enhance its safety.

## Methods and materials

### Ethics statement

All animal experimental procedures were approved by the Institutional Animal Care and Use Committee of Sichuan Agriculture University in Sichuan, China (Protocol Permit Number: SYXK(川)2019-187).

### Cells and viruses

Baby hamster kidney cells (BHK-21) were cultured in Dulbecco’s modified Eagle’s medium (DMEM) (Gibco, Shanghai, China) supplemented with 10% fetal bovine serum (FBS) (Gibco, New York, USA) and incubated at 37 °C with 5% CO_2_.

TMUV strain CQW1 (KM233707.1) was rescued from an infectious clone, which is an early strain isolated in 2013. The epidemic strain CHN-YC (MN966680.1) was isolated in 2019 (gift from Professor Rui Luo, Huazhong Agricultural University). All virus stocks were prepared in BHK-21 cells.

### Infectious clone plasmid construction

We previously reported a full-length infectious clone for the clinical TMUV strain CQW1^25^. All mutants in the present study were generated using this tool with the exception of CQW1-IRES-prME, which was constructed by a DNA-based full-length infectious clone^26^. A diagram of the design of genomes for recombinant TMUV with ectopic expression of structural proteins is shown in Figs. 1a or 6a. The encephalomyocarditis virus internal ribosome entry site (IRES) element and its minimum IRES (MINI) element amplified from the IRES-RLuc-pA plasmid^27^ and foot-and-mouth disease virus (FMDV) 2 A peptide (20 aa in length) were introduced by primers. IRES-mC, MINI-mC, FMDV-2A-mC and IRES-prME were engineered into TMUV cDNA by a series of overlapping PCRs (All of the related primers are presented in Table S1). The IRES or MINI elements are inserted between the termination codon of the NS5 gene and the 3’UTR to generate a bicistronic RNA genome; the FMDV-2A peptide was fused in frame downstream of the NS5 gene. The codons of the ectopic mature capsid (mC) and prM-E genes were all altered to avoid potential RNA recombination events. For viral genomes with ectopic mC, 41-99 aa of CP was deleted in situ. For viral genomes with ectopic prM-E, prM-E was deleted in situ, but the first 10 aa of prM and the last 30 aa of E were retained to ensure the topological structure of the polyprotein on the ER. And the sequences of the infectious cDNA clone for CQW1-IRES-mC and CQW1-MINI-mC have been submitted to GenBank, under the accession number of OM457003 and OM457004, respectively.

### In vitro RNA transcription and RNA transfection

All infectious clone plasmids were purified using a HiPure Plasmid Micro Kit (Magen, Guangzhou, China). The RNA transcription procedure was performed as previously reported^25^. Briefly, the plasmid was linearized using *Sma*I restriction enzyme (NEB, Beijing, China) and was then purified as a template for RNA transcription. An mMESSAGE mMACHINE T7 Transcription kit (Ambion, USA) was used for the transcription of RNA in vitro in a 20 μL reaction with an additional 1.5 μL of GTP solution, which was incubated at 37 °C for 3 h. The DNA template was then removed using DNase I. Afterward, the RNA was purified using the lithium chloride precipitation method, quantitated by spectrophotometry, and stored at –80 °C in aliquots.

For virus rescue, BHK-21 cells were seeded in 12-well plates for 16 h, and the cells (70–90% confluence) were then transfected with 1 μg of RNA per well using Lipofectamine MessengerMAX reagent (Invitrogen, CA, USA). After transfection, the cell plates were incubated at 37 °C with 5% CO_2_. The harvested supernatants from transfected cells (F0 virus) were used to prepare F1 virus stock, and F1 viruses were confirmed by Sanger sequencing.

### Replicon assay and NLuc activity assay

A schematic diagram for a DNA-based replicon is shown in Fig. 2c, and replicon assays were performed as previously reported^28^. WT or mutant replicon plasmids (0.2 μg) were transfected into BHK-21 cells seeded in 96-well plates using TransIntro EL Transfection Reagent (Transgen, Beijing, China). At the indicated timepoint, the cells were washed once with PBS and lysed using Glo lysis buffer (Promega, WI, USA) at room temperature for 5 min. The cell lysates were then directly subjected to the Nluc activity assay or stored at −20 °C.

A Nano-Glo Luciferase Assay System (Promega) was used to detect NLuc activity according to the manufacturer’s instructions. Twenty microliters of the sample and 100 μL of Nano-Glo Luciferase Assay Reagent were added to a white 96-well tissue culture plate and mixed well, and luminescence was detected using a GloMax Navigator System (Promega).

### Indirect immunofluorescence assay (IFA)

IFA was performed as previously reported^16^. Briefly, cells were washed twice with PBS, fixed with 4% paraformaldehyde for 1 h, and then permeabilized for 30 min at 4 °C with 0.3% Triton in PBS. After 1 h of incubation at 37 °C with 5% bull serum albumin (BSA) in PBS, the cells were treated with anti-TMUV mouse polyclonal antibody (self-prepared, 1:200 diluted in PBS containing 1% BSA) for 2 h and then incubated with goat anti-mouse IgG conjugated with FITC (Thermo Fisher Scientific, Shanghai, China; catalog #A16067; 1:1000 dilution) for 1 h. The cells were then stained with DAPI (Coolaber, Beijing, China) in PBS for 10 min. Each step was followed by three 5-min washes with ice-cold PBST (1‰ Tween-20 in PBS) in an orbital shaker. Fluorescence images were acquired under a fluorescence microscope (Nikon, Tokyo, Japan).

### Viral titration, growth curve, and plaque assay

The viral titer was determined using the median tissue culture infective dose (TCID_50_) method with BHK-21 cells. Virus samples were 10-fold serially diluted in DMEM, and then 100 μl dilutions of the viral samples were distributed to each of 8 wells of a 96-well plate seeded with monolayer BHK-21 cells. After 5–7 days incubation at 37 °C, the presence of viruses was determined by observation of CPE using microscopy and viral titers were calculated according to the Karber method.

Viral growth curves on BHK-21 cells seeded in 24-well plates were determined. Cells at 90% confluence were washed twice with PBS and infected with WT virus or mutant virus at a dose of 100 TCID_50_ (MOI = 0.002). After 1.5 h of incubation at 37 °C, the supernatant was removed, and the cells were washed twice and then supplemented with DMEM containing 2% FBS and 1% penicillin/streptomycin. The supernatant was collected every 12 h for viral titration.

The plaque assay was performed as described previously with slight alterations^25^. Briefly, viral samples were serially diluted 10-fold in DMEM; 300 µL of samples of each dilution were distributed to a well of a 12-well plate seeded with nearly confluent BHK-21 cells. After 1.5 h of attachment at 37 °C, the cells were covered with 1 mL of 1% methyl cellulose overlay containing 2% FBS and 1% penicillin/streptomycin. At 5 dpi, the overlay was removed carefully, and the cells were washed twice with PBS, fixed with 4% formaldehyde at room temperature for 20 min, and then stained with 1% crystal violet for 1 min. The cells were washed carefully, and visible plaques were observed.

### Animal experiments

Virulence assays in duck embryos were performed as previously described^25^. Ten 9-day-old duck embryo eggs per group were injected with 100 μL of virus dilution by allantoic cavity inoculation at a dose of 3000 TCID_50_ or 30,000 TCID_50_. The mock group was treated with an equal volume of DMEM. Eggs were then incubated at 37 °C and checked daily with an egg candler. An embryo egg that lost movement and exhibited desquamated blood vessels was regarded as an observation of mortality.

Mouse experiments were performed using 14-day-old suckling mice (Kunming mice). For comparison of the neurovirulence of mutant viruses and WT virus, 8 suckling mice in each group were intracranially injected with 30 µL of WT virus or one of the mutant viruses, respectively, at a dose of 10^4.23^ TCID_50_; those in the mock group was incubated with an equal volume of DMEM. The weight changes, clinical signs and mortalities were monitored and recorded every day, and the mice were raised for 16 days. Mice exhibiting body weight loss of >20% or completely hind legs paralysis were euthanized. The clinical symptoms of the mice were scored according to severity: (1) low spirits and moving slowly, (2) paralysis of the legs, and (3) blindness. For each item, a score of 1 corresponded to 1 mouse showing the corresponding symptom. The total points were calculated as 20% * Item 1 + 50% * Item 2 + 30% * Item 3.

For the duck experiments, we first determined the immune responses of 25-day-old ducks (purchased from Waterfowl Breeding Center of Sichuan Agriculture University) upon infection. Eleven ducks in each group were intramuscularly injected with 200 μL (10^5^ TCID_50_) of the WT virus or one of the mutant viruses, and those in the mock group were incubated with an equal volume of DMEM. At 3 and 5 dpi, 3 ducklings from each group were euthanized (carotid arteries of narcotized ducks were cut open for bloodletting). Then indicated organs were collected to detect the viral loads in tissue samples, including the heart, liver, spleen, lung, kidney, brain, and thymus, by RT–qPCR as previously reported^28^. The remaining ducklings were raised weekly to monitor the neutralizing antibodies in serum. At 14 dpi, the ducks were bled for the T-lymphocyte proliferation assay, and IFN-γ and IL-4 in serum were further analyzed by ELISA.

We then used a sensitive animal model, 5-day-old specific pathogen-free (SPF) ducklings, to evaluate the virulence and preventive effect of the mutant viruses. Ducklings were hatched from SPF duck embryos (purchased from the Harbin Veterinary Research Institute, China) and raised in isolators with negative pressure. Five-day-old ducklings were intramuscularly injected with 200 μL (containing 10^5^ TCID_50_) of the mutant or WT virus. The mock-infected ducklings were given DMEM. The weight changes in ducklings were monitored every two days, and signs of disease and mortality were checked daily. Viremia was determined on BHK-21 cells at 3 dpi. To verify the preventive effect of mutant viruses, ducklings were challenged at 14 dpi with the virulent TMUV strain CHN-YC (at 2 × 10^6^ TCID_50_) by intramuscular injection. On day 3 post-challenge, the ducklings were bled, and viremia was determined. Moreover, the weight changes, clinical symptoms and survival were recorded until 14 days post-challenge. Ducks showing loss of bogy weight more than 20% or stopped gaining body weight for three consecutive days (for animals in growing period), or completely legs paralysis, will be euthanized to reduce the pains. The clinical symptoms of the ducks were scored according to severity: (1) loss of appetite and slow movement, (2) unsteady standing and (3) paralysis of the legs. For each item, a score of 1 corresponded to 1 duck exhibiting the corresponding symptom. The total point was calculated as 20% * Item 1 + 30% * Item 2 + 50% * Item 3.

### T-lymphocyte proliferation assay and detection of the serum levels of IFN-γ and IL-4

The peripheral blood lymphocyte proliferation assay was performed using a modified CCK-8 method as described previously^29^. Briefly, peripheral blood T lymphocytes were isolated using a Peripheral Blood Lymphocyte Separation Kit (Solarbio, Beijing, China) according to the manufacturer’s instructions. After cell counting, 80 μL of diluted cells in RPMI 1640 medium (Gibco) was seeded into a 96-well plate. To specifically stimulate the proliferation of T lymphocytes, 20 µL of CHN-YC virus (10^6.25^ TCID_50_/100 µL) or purified recombinant truncated E protein (20 mg/mL) was added; the unstimulated control was administered an equal volume of PBS. After 36 h of cell culture at 37 °C, cell proliferation was detected using a Cell Counting Kit-8 (MCE, Shanghai, China) according to the manufacturer’s instructions. The stimulation index calculated from the OD value of each stimulated group, and the OD of mock group is normalized to 1.

The levels of Th1-type cytokine IFN-γ and the Th2-type cytokine IL-4 in serum were measured using duck IFN-γ and IL-4 sandwich ELISA kits (ML Bio, Shanghai, China) following the manufacturer’s instructions.

### Plaque reduction neutralization test (PRNT)

The neutralizing antibodies in duckling serum of BHK-21 cells were analyzed by PRNT as described previously^30^. Briefly, serum samples were inactivated at 56 °C for 30 min and continuously diluted 5-fold to 5^−7^ with DMEM. The diluted sera were then mixed with an equal volume of the WT virus (120 TCID_50_); for the virus control groups, an equal volume of DMEM was mixed with the virus. The mixtures were then incubated at 37 °C for 1 h and distributed into 12-well plates seeded with BHK-21 cells. The subsequent procedures of the plaque assay were as described above. The effective dilution of serum to 50% end point titers (NT_50_) was calculated using the Kärber method.

### RNA extraction and real-time quantitative reverse transcription-PCR (RT–qPCR)

The isolation of total RNA and RT–qPCR were performed as previously reported^28^. Briefly, total RNA was isolated using RNAiso Plus (Takara, Dalian, China) according to the manufacturer’s instructions. Subsequently, 1^st^-strand cDNA was transcribed using a HiScript III 1st Strand cDNA Synthesis Kit (Vazyme, Nanjing, China). To measure vRNA copies or the transcriptional expression of cytokines, RT–qPCR assays were performed using 2× Taq SYBR Green qPCR Premix (Innovagene, Changsha, China) with a CFX Connect Real-Time PCR Detect System (Bio–Rad) following the manufacturer’s protocols. All of the related primers for RT–qPCR are presented in Table S1.

### Quantification and statistical analysis

The data from the analyses of NLuc activity and viral titers and the RT–qPCR and ELISA data are presented as the means ± standard errors of the mean (SEMs). Using GraphPad Prism 8.0 software, the statistical significance was assessed by Student’s *t*-test, and significance was defined by a *p*-value < 0.05 (*). The statistical significance of survival was analyzed using a survival curve and the log-rank (Mantel–Cox) test with GraphPad Prism 8.0 software, and significance was defined by a *p*-value < 0.05 (*).

### Reporting summary

Further information on research design is available in the Nature Research Reporting Summary linked to this article.

## Supplementary information

REPORTING SUMMARY

Additional SI

## Data Availability

The data that support the findings of this study are available from the corresponding author upon reasonable request. And the sequences of the infectious cDNA clone for CQW1-IRES-mC and CQW1-MINI-mC have been submitted to GenBank, under the accession number of OM457003 and OM457004, respectively.
