# Supplementary material for: Assembly-defective Tembusu virus ectopically expressing capsid protein is an approach for live-attenuated flavivirus vaccine development
Source: NPJ Vaccines. 2022 May 12;7:51. doi: 10.1038/s41541-022-00468-y (PMC9098475; doi:10.1038/s41541-022-00468-y)
Supplement: Supplementary file 1 — REPORTING SUMMARY [file 41541_2022_468_MOESM1_ESM.pdf]

nature portfolio

Corresponding author(s): DBPR, NPJVACCINES-01762R

Last updated by author(s): Jan 14, 2022

Reporting Summary

Nature Portfolio wishes to improve the reproducibility of the work that we publish. This form provides structure for consistency and transparency in reporting. For further information on Nature Portfolio policies, see our Editorial Policies and the Editorial Policy Checklist.

Statistics

For all statistical analyses, confirm that the following items are present in the figure legend, table legend, main text, or Methods section.

n/a	Confirmed	
☐	☒	The exact sample size (n) for each experimental group/condition, given as a discrete number and unit of measurement
☐	☒	A statement on whether measurements were taken from distinct samples or whether the same sample was measured repeatedly
☐	☒	The statistical test(s) used AND whether they are one- or two-sided<br>*Only common tests should be described solely by name; describe more complex techniques in the Methods section.*
☒	☐	A description of all covariates tested
☒	☐	A description of any assumptions or corrections, such as tests of normality and adjustment for multiple comparisons
☐	☒	A full description of the statistical parameters including central tendency (e.g. means) or other basic estimates (e.g. regression coefficient) AND variation (e.g. standard deviation) or associated estimates of uncertainty (e.g. confidence intervals)
☐	☒	For null hypothesis testing, the test statistic (e.g. F, t, r) with confidence intervals, effect sizes, degrees of freedom and P value noted<br>*Give P values as exact values whenever suitable.*
☒	☐	For Bayesian analysis, information on the choice of priors and Markov chain Monte Carlo settings
☒	☐	For hierarchical and complex designs, identification of the appropriate level for tests and full reporting of outcomes
☒	☐	Estimates of effect sizes (e.g. Cohen's d, Pearson's r), indicating how they were calculated

Our web collection on statistics for biologists contains articles on many of the points above.

Software and code

Policy information about availability of computer code

Data collection	No software was used.
Data analysis	GraphPad Prism 8.4.2 was used to analyze almost all of the data, Bio-Rad CFX Manager 3.0 (gene study) was used to analyze data of RT-qPCR.

For manuscripts utilizing custom algorithms or software that are central to the research but not yet described in published literature, software must be made available to editors and reviewers. We strongly encourage code deposition in a community repository (e.g. GitHub). See the Nature Portfolio guidelines for submitting code & software for further information.

Data

Policy information about availability of data

All manuscripts must include a data availability statement. This statement should provide the following information, where applicable:

- Accession codes, unique identifiers, or web links for publicly available datasets
- A description of any restrictions on data availability
- For clinical datasets or third party data, please ensure that the statement adheres to our policy

The data that support the findings of this study are available from the corresponding author upon reasonable request.

Field-specific reporting

Please select the one below that is the best fit for your research. If you are not sure, read the appropriate sections before making your selection.

☒ Life sciences ☐ Behavioural & social sciences ☐ Ecological, evolutionary & environmental sciences

For a reference copy of the document with all sections, see nature.com/documents/nr-reporting-summary-flat.pdf

Life sciences study design

All studies must disclose on these points even when the disclosure is negative.

Sample size	No sample size calculation, for the present study, at least 3 samples for each independent experiments, and and the sample size is sufficient to access the dependable results, with triplicate measurements in a representative experiment were performed.
Data exclusions	No data were excluded from the study.
Replication	To support the experimental finding of the present study, triplicate measurements in a representative experiment were performed. And all of the experimental are repeatable.
Randomization	The animals were random assorted. Animals were firstly label with mark number, all of mark number of animals were input into a excel sheet, and each animal was distributed a random number by RAND function (=RAND()), then animals were sort by the distributed random number from smallest to biggest, and averagely divide into groups.
Blinding	The present study is not relevant to a clinical study, blinding for collected data is not suitable for the study.

Reporting for specific materials, systems and methods

We require information from authors about some types of materials, experimental systems and methods used in many studies. Here, indicate whether each material, system or method listed is relevant to your study. If you are not sure if a list item applies to your research, read the appropriate section before selecting a response.

Materials & experimental systems

n/a	Involved in the study	
☐	☒	Antibodies
☐	☒	Eukaryotic cell lines
☒	☐	Palaeontology and archaeology
☐	☒	Animals and other organisms
☒	☐	Human research participants
☒	☐	Clinical data
☒	☐	Dual use research of concern

Methods

n/a	Involved in the study	
☒	☐	ChIP-seq
☒	☐	Flow cytometry
☒	☐	MRI-based neuroimaging

Antibodies

Antibodies used	Anti-TMUV polyclony antibody, self-prepared.
Validation	Specificity and validity of Anti-TMUV polyclony antibodies were validated by indirect immunofluorescence assay, which has been well documented in our previously studies.

Eukaryotic cell lines

Policy information about cell lines

Cell line source(s)	BHK-21 cells
Authentication	Cells were authenticated by observing the cells appearance morphology under microscope, and its growth characteristic, sensibility to TMUV virus.
Mycoplasma contamination	The cells were tested negative for mycoplasma contamination by PCR.
Commonly misidentified lines (See ICLAC register)	N/A

Animals and other organisms

Policy information about studies involving animals; ARRIVE guidelines recommended for reporting animal research

Laboratory animals	KUNMING mice, female, two-week-old. Cherry Valley ducks, male/female, 25-day-old. HBK-B duck, male/female, 5-day-old. Cherry Valley ducks embryo, sexless, 9-day-old.
Wild animals	The study did not involve wild animals.
Field-collected samples	The study did not involve samples collected from filed.
Ethics oversight	All animal experimental procedures were approved by the Institutional Animal Care and Use Committee of Sichuan Agriculture University in Sichuan, China (Protocol Permit Number: SYXK([illegible])2019-187).

Note that full information on the approval of the study protocol must also be provided in the manuscript.
